# Differentiation of Clear Cell Renal Cell Carcinoma from other Renal Cell Carcinoma Subtypes and Benign Oncocytoma Using Quantitative MDCT Enhancement Parameters

**DOI:** 10.3390/medicina56110569

**Published:** 2020-10-28

**Authors:** Claudia-Gabriela Moldovanu, Bianca Petresc, Andrei Lebovici, Attila Tamas-Szora, Mihai Suciu, Nicolae Crisan, Paul Medan, Mircea Marian Buruian

**Affiliations:** 1 Department of Radiology and Medical Imaging, Faculty of Medicine, George Emil Palade University of Medicine, Pharmacy, Science and Technology of Târgu Mureș, 540139 Târgu Mureș, Romania; moldovanu_claudia@yahoo.com (C.-G.M.); bianca.petresc@gmail.com (B.P.); mircea.buruian@umfst.ro (M.M.B.); 2 Department of Radiology, Emergency Clinical County Hospital of Cluj-Napoca, 400006 Cluj-Napoca, Romania; 3 Department of Radiology, Faculty of Medicine, Iuliu Hațieganu University of Medicine and Pharmacy, 400012 Cluj-Napoca, Romania; 4 Department of Radiology, Clinical Municipal Hospital, 400139 Cluj-Napoca, Romania; attitamas@yahoo.com; 5 Department of Urology, Clinical Institute of Urology and Kidney Transplant, 400000 Cluj-Napoca, Romania; suciu_umf@yahoo.com; 6 Department of Urology, Faculty of Medicine, Iuliu Hațieganu University of Medicine and Pharmacy, 400012 Cluj-Napoca, Romania; drnicolaecrisan@gmail.com; 7 Department of Urology, Municipal Clinical Hospital, 400139 Cluj-Napoca, Romania; medan.paul@gmail.com; 8 Department of Radiology, Emergency Clinical County Hospital Târgu Mureș, 540136 Târgu Mureș, Romania

**Keywords:** renal cell carcinoma, histological subtypes, oncocytoma, multiphasic multidetector CT (MDCT), quantitative imaging

## Abstract

*Background and objectives*: The use of non-invasive techniques to predict the histological type of renal masses can avoid a renal mass biopsy, thus being of great clinical interest. The aim of our study was to assess if quantitative multiphasic multidetector computed tomography (MDCT) enhancement patterns of renal masses (malignant and benign) may be useful to enable lesion differentiation by their enhancement characteristics. *Materials and Methods*: A total of 154 renal tumors were retrospectively analyzed with a four-phase MDCT protocol. We studied attenuation values using the values within the most avidly enhancing portion of the tumor (2D analysis) and within the whole tumor volume (3D analysis). A region of interest (ROI) was also placed in the adjacent uninvolved renal cortex to calculate the relative tumor enhancement ratio. *Results*: Significant differences were noted in enhancement and de-enhancement (diminution of attenuation measurements between the postcontrast phases) values by histology. The highest areas under the receiver operating characteristic curves (AUCs) of 0.976 (95% CI: 0.924–0.995) and 0.827 (95% CI: 0.752–0.887), respectively, were demonstrated between clear cell renal cell carcinoma (ccRCC) and papillary RCC (pRCC)/oncocytoma. The 3D analysis allowed the differentiation of ccRCC from chromophobe RCC (chrRCC) with a AUC of 0.643 (95% CI: 0.555–0.724). Wash-out values proved useful only for discrimination between ccRCC and oncocytoma (43.34 vs 64.10, *p* < 0.001). However, the relative tumor enhancement ratio (corticomedullary (CM) and nephrographic phases) proved useful for discrimination between ccRCC, pRCC, and chrRCC, with the values from the CM phase having higher AUCs of 0.973 (95% CI: 0.929–0.993) and 0.799 (95% CI: 0.721–0.864), respectively. *Conclusions*: Our observations point out that imaging features may contribute to providing prognostic information helpful in the management strategy of renal masses.

## 1. Introduction

Globally, the incidence of renal cell carcinoma (RCC) varies widely from region to region, with the highest rates being observed in the Czech Republic and North America [1]. According to the revised 2016 World Health Organization classification of RCCs [2], the major subtypes are clear cell RCC (ccRCC), papillary RCC (pRCC), and chromophobe RCC (chrRCC), which comprise 65–70%, 15–20%, and 5–7% of all RCCs, respectively. Oncocytomas are benign lesions that encompass 3% to 5% of renal adult’s neoplasms, and they are the most commonly resected benign renal masses due to misinterpretation at imaging as RCCs [3]. Preoperative classification of RCC into subtypes and recognition of benign lesions has become important because each of them is associated with different treatment choices and prognosis [4,5]. Pre-treatment percutaneous renal mass biopsy is a highly accurate procedure that can be used to identify the histology, differentiate primary from secondary renal masses, and discriminate the less aggressive masses from the more aggressive ones, thus allowing better stratification of patient risk before treatment decisions are made [6]. Lane et al. [7] reported sensitivity for malignancy of 92% and specificity of 90% after more than 2000 renal mass biopsies, with an overall technical failure rate of 5%. Although percutaneous renal mass biopsy remains a valuable method to provide a presurgical histopathologic diagnosis of renal masses, it is an invasive procedure and is not always feasible [8,9]. 

The aim of our study was to assess if quantitative multiphasic multidetector computed tomography (MDCT) enhancement patterns of renal masses (malignant and benign) may be useful to enable lesion differentiation by their enhancement characteristics.

## 2. Materials and Methods

### 2.1. Patients

The Institutional Review Board of Regional Institute of Clinical Municipal Hospital Cluj-Napoca, Romania, approved this retrospective study and waived the requirement for written informed consent (Approval code: Nr. 15/2020; approval date: 11 June 2020). We performed a retrospective analysis in our electronic medical database from January 2017 to June 2020. The inclusion criteria were as follows: patients underwent radical, total, or partial nephrectomy; renal tumor pathologically confirmed (ccRCC, pRCC, chrRCC, oncocytoma); preoperative MDCT with a four-phase renal mass protocol. The exclusion criteria were as follows: other histological types; patients who opted for ablation or active surveillance; the image quality was deemed inadequate due to poor timing of the post-contrast phases or motion artifacts. Finally, 150 patients fulfilled the inclusion criteria with a total of 154 RCC lesions. Three patients had two lesions each (ccRCC subtype), but one had two subtypes of RCC, pRCC in one kidney and ccRCC in the other kidney.

### 2.2. CT Acquisition and Renal Mass Protocol

All CT examinations were performed by using 64—MDCT (Somatom Sensation 64, Siemens, Erlangen, Germany) helical scanner, with the following parameters: 120 kV variable tube current (200–400 mA, depending on patient size), section collimation, 0.6 mm; table feed, 5 mm/sec; and reconstruction interval, 3 mm. The pitch used with helical scanners was 1. The four-phase MDCT renal mass protocol included an unenhanced (UN) scan and contrast-enhanced acquisitions during the corticomedullary (CM), nephrographic (NP), and excretory (EX) phases. Patients received a power injection of nonionic intravenous contrast material into an antecubital vein at a rate of 3.0 mL/s and an infusion dose of 80–150 mL, and a bolus tracking algorithm (CareBolus, Siemens Medical Solutions) was used to determine the onset of imaging in the contrast-acquisitions phases. For bolus tracking, a region of interest (ROI) was placed in the thoracoabdominal aorta junction, with a trigger set to begin at 150 HU, and CM phase imaging occurred 30 s, NP phase imaging occurred 90 s, and EX phase imaging occurred 8 min after the threshold level was reached. 

### 2.3. CT Image Analysis

Image analysis was performed by two radiologists, a radiology resident (C.-G.M.) with 4 years of experience in agreement with a senior radiologist with 8 years of experience in the urogenital field (A.T.-S.). All images were reviewed using a workstation monitor for image archiving and the communication system (KODAK Carestream Version 10.2). The two radiologists were blinded by the pathological results. 

**Figure 1:**
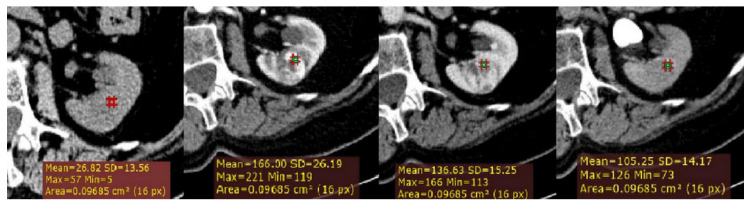
Example of quantitative enhancement measurements from a 72-year-old man with pathological assessment proven clear cell renal cell carcinoma (ccRCC): one circular region of interest (ROI) (0.1 cm^2^ in size) was manually selected in the maximally enhancing portion of tumor by visual inspection in each imaging phase (green ROIs).

Firstly, a ROI cursor approximately 0.1 cm^2^ in size (2D ROI) was placed in the same location of the tumor on each of the four imaging phases of MDCT in the axial plane (Figure 1). For homogeneous lesions, ROIs were placed in the center of the lesion, and for heterogeneous lesions, the ROIs were placed in the maximally enhancing portion of the solid tumor by visual inspection in each imaging phase, excluding the areas of necrosis, calcification, cystic, or hemorrhagic. Thus, we calculated for each lesion the following measurements: the absolute peak lesion enhancement (CM phase (HU)—UN phase (HU)), absolute peak lesion de-enhancement (CM to NP (CM phase (HU)—NP phase (HU)); and NP to EX (NP phase (HU)—EX phase (HU)); and absolute peak lesion enhancement wash-out (%) using a CT formula developed by Kopp et al. [10]. 

**Figure 2:**
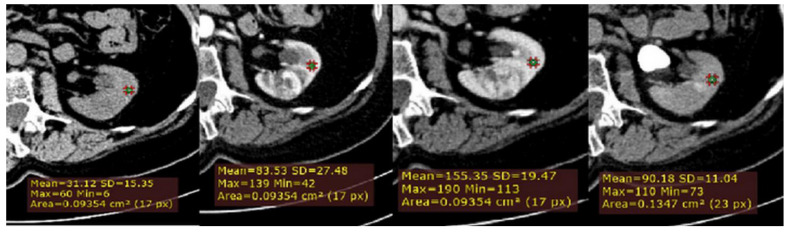
Example of quantitative enhancement measurements from a 72-year-old man with pathological assessment proven ccRCC: a second circular ROI was placed in the adjacent uninvolved renal cortex in each phase (green ROIs).

Secondly, to normalize for variation in attenuation due to individual patient and technical factors, a ROI cursor (0.1 cm2 in size) was placed in the same location of adjacent uninvolved renal cortex, on each of the four imaging phases of MDCT in the axial plane (Figure 2). Next, we calculated for all lesions, the relative tumor enhancement ratio for the CM, NP and EX phase, as follows: (HU tumor enhancement in the postcontrast phase–HU tumor in the unenhanced phase)/(cortex enhancement in the postcontrast phase–cortex in the unenhanced phase).

**Figure 3:**
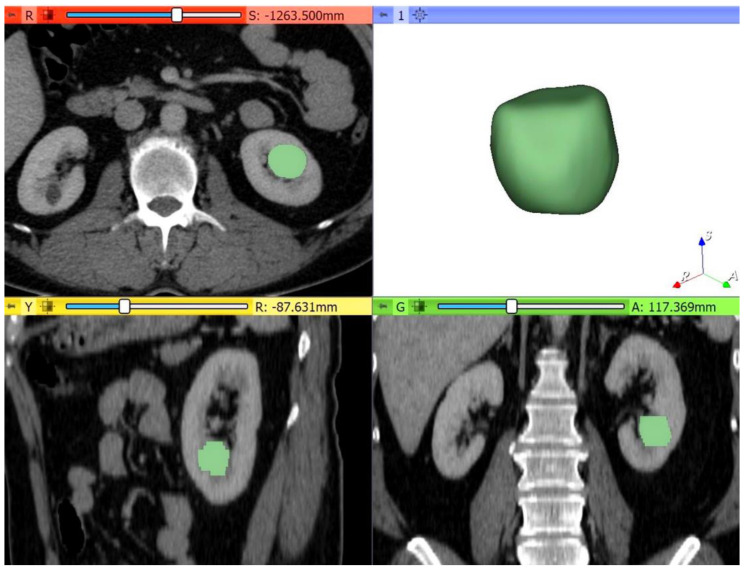
Example of quantitative enhancement measurements from a 52-year-old woman with pathological assessment proven ccRCC: the entire tumor volume was manually contoured in the axial plane in each of the four phases resulting in a 3D tumor volume of interest (VOI) representative of the entire mass (green color = tumoral mass).

Thirdly, the multiphase MDCT acquisitions were exported from the picture archiving and communication system (PACS, Carestream, Concord, ON, Canada) and then transferred to an independent workstation for segmentation using an open-source software 3D Slicer, version 4.10.2 (www.slicer.org). Thus, all renal masses were manually segmented, slice by slice, to obtain a three-dimensional (3D) volume of interest (VOI) over the entire tumor (Figure 3). The nephrographic phase was used for segmentation as it provided the adequate demarcation between the tumor and the normal parenchyma. Contouring was carefully drawn within the borders of the tumors, including necrotic, cystic changes, and hemorrhagic areas. The 3D tumor VOI was used as the ROI to calculate the volume enhancement, de-enhancement (HU), and wash-out (%) values over the whole tumor. The same formulas were used for the peak lesion measurements mentioned above.

### 2.4. Interobserver Reproducibility

The interobserver reliability of the attenuation values from two different ROI groups was evaluated with the intraclass correlation coefficient (ICC). The measurements were performed independently by another senior radiologist (with 8 years of experience in urogenital imaging, A. L.), also blinded by the pathological results. Quantitative enhancement measures with ICC values equal to or greater than 0.75 indicating good reproducibility were included for further analysis.

### 2.5. Statistical Analysis

Continuous variables with normal distribution were expressed as means ± standard deviation. Normality was tested with the Kolmogorov–Smirnov test. To compare the magnitude of enhancement, de-enhancement, and the % wash-out between ccRCCs and pRCCs, chrRCCs, and benign oncocytomas, we performed ANOVA with post hoc analysis (Dunnett T3 tests).

We also performed ANOVA with post hoc analysis (Dunnett T3 tests) to compare the magnitude of enhancement in the adjacent uninvolved renal cortex among the four histological types from each phase. Receiver operating characteristic (ROC) curves were computed, and corresponding areas under the ROC curves (AUCs) were calculated to compare the diagnostic performance of each independent parameter and the prediction model, respectively. To summarize the potential utility of the features, we calculated sensitivity, specificity, cut-off values, and the corresponding 95% confidence intervals (CI). Categorical variables were analyzed by χ2 test. A *p* value of <0.05 was considered statistically significant. All statistical analyses were performed using MedCalc for Windows, version 14.8 (MedCalc Software, Ostend, Belgium) and SPSS Statistics for Windows, version 18.0 (SPSS Inc., Chicago, IL, USA).

## 3. Results

A total of 150 patients were retrospectively included in this study (mean age 60 years, ±12.4 (standard deviation); range 30–84 years), including 98 men (mean age, 59 years, ±12.3; age range 30–84 years) and 52 women (mean age, 62 years, ±12.6; age range 30–83 years) with 154 renal masses. Of these, 123 were ccRCCs, 10 were pRCCs, 10 were chrRCCs, and 11 were oncocytomas. The clinicopathologic characteristics of our study population are summarized in Table 1. 

**Figure 4:**
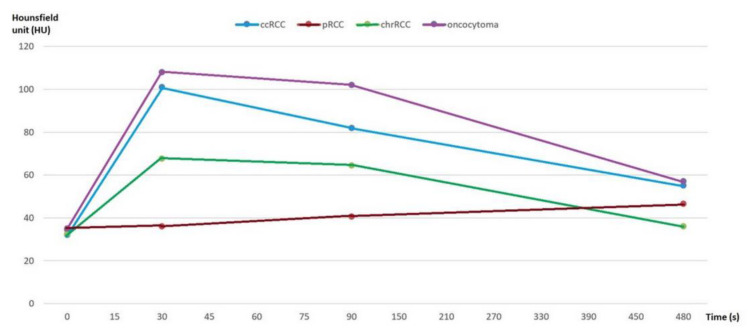
Multiphasic attenuation curves for ccRCCs (*n* = 123), pRCCs (*n* = 10), chrRCCs (*n* = 10), and benign oncocytomas (*n* = 11). Data points are mean attenuation for each phase. Time: 0 s (UN phase), 30 s (CM phase), 90 s (NP phase), and 480 s (EX phase).

First, we analyzed whether the renal masses enhance differently after contrast administration, both for the values obtained by 2D analysis and by 3D analysis. We found that, after contrast administration, oncocytoma had the highest enhancement change, and among the subtypes of RCC, ccRCC displays the highest enhancement, whereas chrRCC enhances moderately and pRCC enhances the least (Figure 4).

Then, using the formulas mentioned above, quantitative enhancement MDCT measures were compared between ccRCC and other types of renal masses (pRCC, chrRCC, and oncocytoma). According to the standard of the ICC > 0.75 in the interobserver tests, we selected for further analysis only the measurements with good reproducibility. Of the quantitative enhancement MDCT measurements that were compared, we found high reproducibility at most values, except for a few: absolute peak lesion enhancement wash-out values, relative tumor enhancement ratio (EX phase), and 3D tumor volume de-enhancement (CM-NP phase). 

We performed univariate analysis to determine if there are any differences between quantitative MDCT parameters and renal lesions (Table 2). Our results show a significant difference in the absolute peak lesion enhancement values between ccRCCs and pRCCs (62.28 vs. 3.40, *p* < 0.001), and ccRCCs and chrRCCs, respectively (62.28 vs. 35.10, *p* = 0.010). Furthermore, the values of absolute peak lesion de-enhancement from the NP to EX phase were significantly different among ccRCCs compared with pRCCs (27.04 vs. −5.10, *p* < 0.020) and oncocytomas, respectively (27.04 vs. 50.64, *p* = 0.022). However, we did not observe significant differences in the absolute peak lesion de-enhancement values from the CM to NP phase between ccRCC and other types of renal masses. 

3D tumor ROI enhancement measurements were significantly different between ccRCCs and pRCCs (44.86 vs. 9.80, *p* < 0.001), between ccRCCs and chrRCCs (44.86 vs. 30.20, *p* = 0.046), and between ccRCCs and oncocytomas, respectively (44.86 vs. 74.36, *p* = 0.010). 3D tumor ROI de-enhancement values from the NP phase to the EX phase were also found to be significantly different between ccRCCs and pRCCs (21.93 vs. 3.20, *p* = 0.022), and ccRCCs and oncocytomas, respectively (21.93 vs. 44.64, *p* = 0.005). 3D tumor ROI wash-out measurements were only significant between ccRCCs and oncocytomas (43.34 vs. 64.10, *p* < 0.001). 

As suggested previously [11], the measured attenuation of the renal masses should be normalized by using the measured attenuation of the uninvolved renal cortex to ensure that attenuation is independent of the patient or technical variability. Our findings show that the relative tumor enhancement ratio was significantly in the CM phase between ccRCCs and pRCCs (0.97 vs. 0.02, *p* < 0.001), and chrRCCs, respectively (0.97 vs. 0.36, *p* < 0.001), and in the NP phase between ccRCCs and pRCCs (0.57 vs. 0.01, *p* = 0.003), and chrRCCs, respectively (0.57 vs. 0.34, *p* = 0.024).

**Figure 5:**
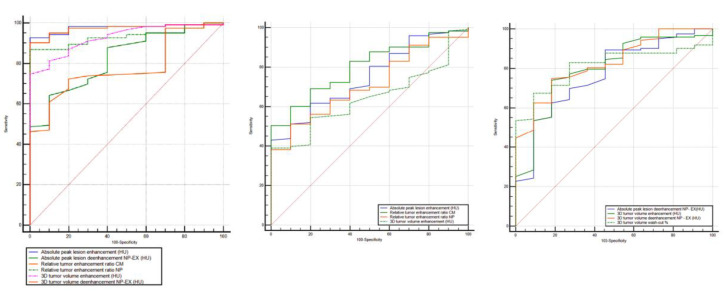
Receiver operating characteristic (ROC) curves for the combination of quantitative features for differentiation of ccRCC from other RCC subtypes (pRCC and chrRCC) and benign oncocytoma. RCC, renal cell carcinoma; AUC, area under the ROC curve; NP, nephrographic phase; EX, excretory phase.

Furthermore, to discriminate ccRCC from pRCC, chrRCC, and oncocytoma, all the statistically significant different measurements resulted in AUCs > 0.6 (Figure 5, Table 3). To differentiate between ccRCC and pRCC, absolute peak lesion enhancement measurement demonstrated the highest AUC of 0.976 (95% confidence interval (CI): 0.924–0.995) with 92.7% sensitivity and 100% specificity, when using 17 HU as the cutoff value. Between ccRCC and chrRCC, relative tumor enhancement ratio CM phase showed the highest AUC of 0.799 (95% CI: 0.721–0.864) with 50.4% sensitivity and 100% specificity, when using 0.72 as the cutoff value. Regarding the differentiation between ccRCC and oncocytoma, 3D tumor ROI de-enhancement NP to EX phase measurement had the highest AUC of 0.827 (95% CI: 0.752–0.887) with 74.8% sensitivity and 81.8% specificity, when using 33 HU as the cutoff value.

## 4. Discussion

Although the goals of radiologic imaging are to detect and stage renal tumors, in the last decade there has been a substantial clinical interest for preoperative classifications of renal masses subtypes using MDCT quantitative assessments. Since clinical implications and therapeutic strategies may differ for subtypes of renal cortical tumors, the development of noninvasive techniques and markers to predict the histological type of renal masses would be of great clinical interest. The noninvasive characterization of renal masses with images would have some advantages: no additional cost to the patient, no subsequent appointment for additional testing, no added direct procedure-related risk, and it can be used to evaluate the whole tumor (especially important when a heterogeneous tumor is characterized) [12,13,14]. 

Our study is one of the few that included 3D tumor ROI for the analysis of attenuation enhancement values. Using a 3D tumor ROI can avoid inter- and intra-observer variability associated with manual placement of ROI [15], especially in heterogeneous tumors [16]. 

Like the studies conducted by Bird et al. [17] and Zhang et al. [18], we found that after contrast administration, oncocytoma showed the highest enhancement change, and among the subtypes of RCC, ccRCC displayed the highest enhancement, whereas chrRCC enhanced moderately and pRCC enhanced the least. In our study, these results were obtained both when we used a 2D ROI in the most avidly enhancing portion of the tumor and also when we used a 3D ROI representative for the entire tumor to quantify whole lesion enhancement. However, research by Young et al. [19] is in contradiction with these results, which found that the magnitude of the enhancement of ccRCC was significantly greater than that of pRCC, chrRCC, and oncocytoma in all postcontrast phases. This may be due to the different designs of the studies. Young et al. included patients who were not scanned on the same scanner with the same four-phase MDCT protocol and they used subjectively selected smaller ROIs from 2D images so that each level of the enhancement threshold was assessed for statistical analysis.

Most papers [20,21,22,23,24,25,26,27,28,29] that have analyzed the association between attenuation values on MDCT and histological types have shown that the magnitude of enhancement at MDCT can help differentiate ccRCC from pRCC, chrRCC, and oncocytoma. Using measurements of quantitative MDCT enhancement values, our study showed that, among solid cortical renal tumors, the greatest utility in terms of differentiations was between ccRCC and pRCC. The ROC analysis showed that absolute peak lesion enhancement values (2D analysis) can discriminate ccRCC and pRCC with the highest AUC of 0.976 (95% CI: 0.924–0.995) when using 17 HU as the cutoff value. Regarding the values of the measurements obtained from 3D ROI (over the whole tumor), our work demonstrates that between ccRCC and pRCC, significant differences are obtained both for the values of volume enhancement, de-enhancement (HU), and wash-out (%). Research by Chen et al. [30] also assessed whole lesion quantitative enhancement parameters, proving significant differences between ccRCC and pRCC on all postcontrast phases.

Kopp et al. [10] proposed a CT wash-out formula for differentiating ccRCC from other renal masses. Their study found rapid wash-out in ccRCC and they did not observe this to be significantly different from that seen in oncocytoma. Due to the fact that they did not include CM phase in the MDCT protocol in their study, including only UN, NP, and EX phases. In our study, we used this proposed formula using a four-phase MDCT protocol to calculate wash-out values. When we used 2D ROIs we did not observe significant differences between ccRCC and the other renal masses, but when we used 3D ROIs, we found that the measurements were only significant between ccRCCs and oncocytomas (43.34 vs. 64.10, *p* < 0.001). 

Another paper [24] proposed an enhancement correcting method in order to differentiate the renal carcinomas on MDCT. They used a formula created to obtain attenuation values that are corrected to a certain standard in the aorta at the level of the organ-supplying vessel. The results of this paper proved that the differentiation of ccRCC from pRCC, using the corrected attenuation in the CM and NP phase, was accurate (95.7% and 94.8%, respectively), with a cutoff value of 100 HU in the CM phase and 85 HU in the NP phase. In our opinion, this method is useful when evaluating the measurements of ROIs on the most avidly enhancing portions of the tumor, but it is hard to reproduce when assessing the measurements of ROIs on the whole lesion enhancement.

The research of Zhang et al. [18], using a three-phase MDCT protocol, did not find a difference in enhancement between ccRCCs and oncocytomas. This is in contradiction with our results, we found differentiation between ccRCC and oncocytoma in the enhancement, de-enhancement, and wash-out values. Measurement of 3D tumor ROI de-enhancement NP to EX phase had the highest AUC of 0.827 (95% CI: 0.752–0.887) with 74.8 % sensitivity and 81.8 % specificity when using 32 HU as the cutoff value.

Regarding the relative enhancement parameter, the research of Ruppert-Kohlmayr et al. [24] demonstrated a significant difference between ccRCC and pRCC both in the CM phase and in the NP phase (*p* < 0.05). They reported that ccRCC had higher values than the cutoff value in CM (of 2.0) and NP phase (of 1.8), whereas pRCC had lower values than the cutoff value in the CM and NP phase. Findings in this paper are partially supported by our study; we found a lower cutoff value in the CM ad NP phase of 0.21 and 0.24, respectively. This may be because of two key differences between our study and theirs. Firstly, Ruppert-Kohlmayr et al. used a three-phase MDCT protocol (UN, CM, and NP phase), whereas we used a four-phase MDCT protocol (UN, CM, NP, and EX phase). Secondly, Ruppert-Kohlmayr et al. used another formula for calculating the relative enhancement of the lesions than the one we used. They defined the relative enhancement ratio as the ratio between the corrected attenuation in a contrast phase and the measured attenuation in an unenhanced phase. In addition, we used a formula proposed by Coy et al. [15]: (HU tumor enhancement in the postcontrast phase—HU tumor in the unenhanced phase)/(cortex enhancement in the postcontrast phase—cortex in the unenhanced phase). 

The present study has some limitations. The major one was the relatively small number of lesion subtypes. Further studies would be needed to demonstrate these promising results. Secondly, due to its retrospective nature, it could have a selection bias. Thirdly, the results depended on where the ROIs are drawn and, therefore, a standardized method of ROI measurement should be developed. Assessment of whole lesion attenuation values of renal lesions is technically more challenging than 2D ROI-based assessment of renal tumors, and may not be feasible in all clinical practice. Moreover, it is also not yet clear whether the assessment of the whole lesion is more accurate than 2D ROI-based assessment. Therefore, further studies are needed to compare the assessment of the whole lesion and the ROI assessment of renal cell carcinoma, both for accuracy and ease of use. Another limitation is that the four-phase MDCT renal mass protocol results in a high patient radiation dose and should be reserved only for cases in which lesion discrimination is required before treatment selection. Moreover, many of the renal tumors resected at our institution (approximately 400 lesions) did not have preoperative multiphasic MDCT scans available for review and were not included in our analysis. Even with these potential limitations, the results of our analyses suggest that there may be a consistent relationship between enhancement at MDCT and renal tumor histologic findings. However, these results should be validated in a large cohort, preferably in a prospective manner.

## 5. Conclusions

Based on our findings, we believe that quantitative MDCT enhancement patterns can help distinguish ccRCC from malignant RCC subtypes and benign oncocytoma. Given our findings, quantitative MDCT enhancement patterns may be a preliminary step in the development of a multiparametric decision model that can serve as an adjunct in clinical decision making for proper management.

## Figures and Tables

**Table 1:** Characteristics of patients and renal lesions. Data are the number of patients (*n* = 150) and data in parentheses are percentages, except where otherwise indicated. * Data in parentheses are the range.

Characteristic	Clear Cell RCC *n* = 123 (79)	Papillary RCC *n* = 10 (6)	Chromophobe RCC *n* = 10 (6)	Oncocytoma *n* = 11 (7)
**Gender**				
Male	85 (55)	7 (5)	5 (3)	5 (3)
Female	38 (25)	3 (3)	5 (3)	6 (4)
Mean age (y) *	61 (30–84)	61 (49–79)	54 (34–71)	56 (34–73)
**Method of specimen acquisition**				
Partial nephrectomy	21 (14)	2 (2)	1 (1)	5 (3)
Radical nephrectomy	56 (36)	6 (4)	4 (3)	4 (3)
Total nephrectomy	46 (30)	2 (2)	5 (3)	2 (1)
**Pathologic tumor stage**				
T1a	31 (20)	3 (3)	1 (1)	-
T1b	29 (19)	1 (1)	4 (3)	-
T2a	11 (7)	3 (3)	1 (1)	-
T2b	3 (3)	0 (0)	1 (1)	-
T3a	32 (21)	3 (3)	1 (1)	-
T3b	13 (8)	0 (0)	2 (2)	-
T4	1 (1)	0 (0)	0 (0)	-
**Fuhrman grade**				
I	26 (17)	2 (2)	1 (1)	-
II	62 (40)	6 (4)	8 (5)	-
III	27 (18)	2 (1)	1 (1)	-
IV	8 (5)	0 (0)	0 (0)	-
**Side**				
Left	59 (38)	4 (3)	5 (3)	7 (5)
Right	59 (38)	6 (4)	5 (3)	4 (3)
Both kidneys	3 (3)	0 (0)	0 (0)	0 (0)
**Lesion size (cm)**				
<4	32 (21)	6 (4)	1 (1)	7 (5)
4–7	41 (27)	2 (2)	6 (4)	4 (3)
7–10	37 (24)	1 (1)	1 (1)	0 (0)
>10	13 (8)	1 (1)	2 (1)	0 (0)

**Table 2:** Quantitative enhancement characteristics of renal masses of the study population. Data are the mean and 95% CI of the mean in parentheses. * *p* value < 0.05 was considered statistically significant.

Enhancement Characteristic	Clear Cell RCC	Papillary RCC	Chromophobe RCC	Oncocytoma
Absolute peak lesion enhancement (HU)	67.28 (60.49–74.06)	3.40 (−3.45–10.25)	35.10 (18.17–52.03)	81.36 (56.08–106.65)
*p* vs. clear cell RCC		<0.001 *	0.010 *	0.792
*p* vs. papillary RCC	<0.001 *		0.012 *	<0.001 *
*p* vs. chromophobe RCC	0.010 *	0.012 *		0.019 *
*p* vs. oncocytoma	0.792	<0.001 *	0.019 *	
Absolute peak lesion de-enhancement (HU)				
Corticomedullary to nephrographic	17.67 (13.02–22.31)	−1.20 (−16.01–13.61)	5.00 (−4.55–14.55)	10.18 (−16.95–37.32)
*p* vs. clear cell RCC		0.101	0.101	0.988
*p* vs. papillary RCC	0.101		0.958	0.951
*p* vs. chromophobe RCC	0.101	0.958		0.999
*p* vs. oncocytoma	0.988	0.951	0.999	
Nephrographic to excretory	27.04 (22.78–31.30)	−5.10 (−23.94–13.74)	23.80 (16.76–30.84)	50.64 (36.55–64.72)
*p* vs. clear cell RCC		<0.020 *	0.943	0.022 *
*p* vs. papillary RCC	<0.020 *		0.040 *	<0.001 *
*p* vs. chromophobe RCC	0.943	0.040 *		0.010 *
*p* vs. oncocytoma	0.022 *	<0.001 *	0.010 *	
Relative tumor enhancement ratio				
Corticomedullary phase	0.97 (0.78–1.17)	0.02 (−0.07–0.12)	0.36 (0.19–0.53)	0.76 (0.49–1.02)
*p* vs. clear cell RCC		<0.001 *	<0.001 *	0.655
*p* vs. papillary RCC	<0.001 *		0.008 *	<0.001 *
*p* vs. chromophobe RCC	<0.001 *	0.008 *		0.069
*p* vs. oncocytoma	0.655	<0.001 *	0.069	
Nephrographic phase	0.57 (0.50–0.63)	0.01 (−0.23–0.267)	0.34 (0.20–0.47)	0.77 (0.42–1.11)
*p* vs. clear cell RCC		0.003 *	0.024 *	0.743
*p* vs. papillary RCC	0.003 *		0.119	0.005 *
*p* vs. chromophobe RCC	0.024 *	0.119		0.112
*p* vs. oncocytoma	0.743	0.005 *	0.112	
3D tumor volume enhancement (HU)	44.86 (40.08–49.64)	9.80 (4.88–14.72)	30.20 (20.67–39.73)	74.36 (58.65–90.07)
*p* vs. clear cell RCC		<0.001 *	0.046 *	0.010 *
*p* vs. papillary RCC	<0.001 *		0.005 *	<0.001 *
*p* vs. chromophobe RCC	0.046 *	0.005 *		<0.001 *
*p* vs. oncocytoma	0.010 *	<0.001 *	<0.001 *	
3D tumor volume de-enhancement (HU)				
Nephrographic to excretory	21.93 (18.86–25.01)	3.20 (−7.91–14.31)	13.60 (7.00–20.20)	44.64 (33.51–55.77)
*p* vs. clear cell RCC		0.022 *	0.125	0.005 *
*p* vs. papillary RCC	0.022 *		0.395	<0.001 *
*p* vs. chromophobe RCC	0.125	0.395		<0.001 *
*p* vs. oncocytoma	0.022 *	<0.001 *	<0.001*	
3D tumor volume enhancement wash-out (%)	43.34 (37.79–48.89)	−5.33 (−73.72–63.05)	38.97 (15.25–62.69)	64.10 (58.71–69.48)
*p* vs. clear cell RCC		0.540	0.999	<0.001 *
*p* vs. papillary RCC	0.540		0.672	0.220
*p* vs. chromophobe RCC	0.999	0.672		0.199
*p* vs. oncocytoma	<0.001 *	0.220	0.199	

**Table 3:** ROC curves for combination of quantitative features for differentiation of ccRCC from other RCC subtypes (pRCC and chrRCC) and benign oncocytoma. RCC, renal cell carcinoma; AUC, area under the ROC curve; NP, nephrographic phase; EX, excretory phase; * *p* value < 0.05 was considered statistically significant.

Discrimination of Clear Cell RCC	AUC (95%CI)	* *p* Value	Sensitivity (95%CI)	Specificity (95%CI)	Cutoff Value
From papillary RCC					
Absolute peak lesion enhancement	0.976 (0.924–0.995)	<0.001	92.7 (86.6–96.6)	100 (69.2–100)	17
Absolute peak lesion de-enhancement NP to EX phase	0.825 (0.750–0.886)	<0.020	64.2 (55.1–72.7)	90 (55.5–99.7)	15
Relative tumor enhancement ratio CM phase	0.973 (0.929–0.993)	<0.001	90.2 (83.6–94.9)	100 (69.2–100)	0.21
Relative tumor enhancement ratio NP phase	0.931 (0.874–0.968)	0.003	87 (79.7–92.4)	100 (69.2–100)	0.24
3D tumor volume enhancement	0.928 (0.871–0.966)	<0.001	74.8 (66.2–82.2)	100 (79.2–100)	22
3D tumor volume de-enhancement NP to EX phase	0.778 (0.698–0.846)	0.022	72.4 (63.6–80)	80 (64.2–97.5)	10
From chromophobe RCC					
Absolute peak lesion enhancement	0.759 (0.668–0.821)	0.010	43.1 (34.2–52.3)	100 (69.2–100)	71
Relative tumor enhancement ratio CM phase	0.799 (0.721–0.864)	<0.001	50.4 (41.2–59.5)	100 (69.2–100)	0.72
Relative tumor enhancement ratio NP phase	0.711 (0.626–0.787)	0.024	51.2 (42.0–60.3)	90.0 (55.5–99.7)	0.54
3D tumor volume enhancement	0.643 (0.555–0.724)	0.046	39 (30.4–48.2)	100 (69.2–100)	51
From oncocytoma					
Absolute peak lesion de-enhancement NP to EX phase	0.771 (0.690–0.839)	0.022	53.7 (44.4–62.7)	90.9 (68.7–99.8)	32
3D tumor volume enhancement	0.798 (0.720–0.862)	0.010	74.0 (65.3–81.5)	81.8 (68.2–97.7)	67
3D tumor de-enhancement NP to EX phase	0.827 (0.752–0.887)	0.005	74.8 (66.2–82.2)	81.8 (68.2–97.7)	33
3D tumor volume wash-out	0.798 (0.720–0.862)	<0.001	74 (65.3–81.5)	81.8 (48.2–97.7)	67
